# Incidental findings of borderline ovarian tumor or ovarian cancer – real-world data on surgical and oncological outcomes

**DOI:** 10.3389/fonc.2024.1450461

**Published:** 2024-10-11

**Authors:** Carmen Joder, Celine Smaadahl-Wey, Lara Zumwald, Flurina Saner, Claudia Rauh, Seline Hofer, Julian Wampfler, Saskia Schlootz, Tilman Rau, Lucine Christe, Wiebke Solass, Sara Imboden, Michael David Mueller, Franziska Siegenthaler

**Affiliations:** ^1^ Faculty of Medicine, University of Bern, Bern, Switzerland; ^2^ Department of Obstetrics and Gynecology, Bern University Hospital and University of Bern, Bern, Switzerland; ^3^ Department of Medical Oncology, Bern University Hospital, Bern, Switzerland; ^4^ Institute of Tissue Medicine and Pathology, University of Bern, Bern, Switzerland

**Keywords:** ovarian cancer, borderline ovarian tumor, centralized care, surgical cytoreduction, surgical morbidity, oncological outcome

## Abstract

**Introduction:**

Centralization of ovarian cancer treatment is associated with higher rates of optimal surgery and longer survival. However, preoperative diagnosis of ovarian cancer is challenging and some diagnoses are made incidentally after surgery. This study investigated the surgical and oncological outcomes of patients with incidental findings of borderline ovarian tumors or ovarian cancer who were centralized postoperatively and treated with a two-stage surgical procedure, and compared these with those of patients with adnexal masses of suspected malignancy who were offered a single-stage surgical procedure with intraoperative frozen section in a tertiary hospital.

**Methods:**

A database of 390 patients with adnexal masses and surgical treatment at the Bern University Hospital, Switzerland was retrospectively reviewed to identify patients with borderline ovarian tumors or ovarian cancer between 2010 and 2020.

**Results:**

Among 390 patients with adnexal masses, 223 were diagnosed with a borderline ovarian tumor or ovarian cancer. Compared with patients with suspected malignancy and a centralized single-stage surgical procedure, patients with an incidental postoperative malignancy diagnosis and a two-stage surgical procedure underwent more surgical interventions (1.3 vs. 2.1 p<.001) and had a longer time interval from diagnosis to initiation of chemotherapy (33.3 vs. 45.1 p=.005) and to completion of surgical cytoreduction (31.9 vs. 73.7 days, p<.001). However, there were no differences in the rates of complete cytoreduction (90.0% vs. 93.2%, p=.719), intraoperative (11.3% vs. 13.7%, p=.664) or postoperative (38.7% vs. 37.0%, p=.884) complication rates, and number of hospitalization days (11.1 vs. 12.0 days, p=.369). An incidental diagnosis of malignancy with postoperative referral was neither associated with an increased risk of recurrence (hazard ratio (HR) 0.8, 95% confidence interval (CI) 0.6-1.8, p=.839) nor death (HR 0.7, 95% CI 0.4-1.1, p=.113), and there was no difference in mean recurrence-free survival between the study subgroups.

**Discussion:**

Although patients with incidental findings of borderline ovarian tumors or ovarian cancer treated with a two-stage surgical procedure had a longer time to completion of surgical staging and initiation of chemotherapy, our results showed no negative impact on oncological outcomes.

## 1 Introduction

Ovarian cancer is the most lethal gynecological malignancy in the developed world and given its insidious growth, the delayed onset of symptoms, and the lack of proper screening methods, it is often diagnosed at an advanced stage (1, 2). To ensure optimal disease management, centralization of care for high-grade ovarian cancer has been recommended by the European Society of Gynecological Oncology (ESGO) in 2016 (3). Centralization improves access to hospitals with higher surgical volume and advanced technologies and therapies, to gynecologic oncologists with a high level of surgical expertise, and to multidisciplinary cancer teams (4–7). Several studies have shown that these factors contribute to improved staging, more standardized treatments, more patient-focused care with complex surgeries, and an increase in complete cytoreduction, ultimately leading to better survival outcomes (3–9). However, since preoperative diagnosis of ovarian cancer remains difficult and some diagnoses are made unexpectedly after final pathology, it becomes challenging to provide centralized care to all patients presenting with adnexal masses of uncertain malignancy (10–12). Given that adnexal masses are extremely common in women, yet only a small proportion of them turn out to be ovarian cancer or borderline ovarian tumors, it is questionable whether all patients with adnexal masses need to be referred to a tertiary hospital prior to diagnostic surgery to ensure optimal disease management. Our study examined this issue by comparing the surgical and oncological outcomes of patients who underwent a single-stage surgery with intraoperative frozen section at a tertiary center with those who underwent a second surgery at a tertiary center after incidental finding of malignancy at a non-tertiary hospital.

## 2 Materials and methods

### 2.1 Patient population

A retrospective observational study was conducted of patients with adnexal masses who underwent surgical treatment at the ESGO certified cancer center of the University Hospital of Bern, Switzerland, between 2010 and 2020. Eligible participants were aged ≥18 years with diagnosis of borderline ovarian tumors or ovarian cancer who were either referred preoperatively to our tertiary institution for suspected malignancy (group 1) or who underwent initial surgery at a non-tertiary center and were referred postoperatively to our institution for incidental malignancy findings to complete surgical staging and for adjuvant treatment (group 2). Patients with a benign histology or metastases as adnexal mass were excluded. Clinicopathological data were retrieved from an electronic database. The local ethics committee Bern, Switzerland approved the study protocol and all patients signed written informed consent (reference number 2018-00479).

### 2.2 Diagnostics

Diagnostic parameters collected included preoperative imaging and mean preoperative CA 125 levels.

### 2.3 Treatment

Treatment parameters collected were use of chemotherapy and surgical cytoreduction, including mean time from diagnosis to initiation of treatment, number of surgical procedures and hospitalization days, complications, and optimal cytoreduction. Patients in group 1 were referred preoperatively to our tertiary hospital. They were offered a single-step surgical procedure including intraoperative frozen section analysis. If complete cytoreduction or staging was feasible, it was performed during the same surgical procedure. Otherwise, they received neoadjuvant chemotherapy and were re-evaluated after 3 cycles. In these patients, a diagnostic laparoscopy was performed to obtain histological confirmation and to assess the extent of the disease. Patients in group 2 underwent diagnostic surgery at a non-tertiary hospital and were referred postoperatively to our tertiary hospital for surgical cytoreduction after detection of malignancy by final pathology. Possible reasons for incidental diagnosis of malignancy in group 2 included suspected adnexal torsion, incidental finding of adnexal cyst in imaging, or suspected benign mass. Treatment decisions were based on national and international guidelines (13, 14). Follow-up data on recurrence and survival were available through standardized databases and follow-up controls.

### 2.4 Clinical outcome

Clinical outcome parameters collected were risk of recurrence or death, overall survival, and recurrence-free survival. Recurrence-free survival was defined as time from primary staging surgery to first recurrence or death of any cause. Overall survival was calculated from date of primary staging surgery until death or until the date of the last follow-up.

### 2.5 Statistical analysis

Statistical analysis was performed using the Statistical Package for Social Sciences (IBM SPSS Statistic version 28.0.1.1). Categorical variables were reported as frequencies and proportions, while continuous variables were reported as means and standard deviations. Formal comparisons were made using Chi-square statistics (χ2) or Fisher’s exact test for categorical variables and T-test or analysis of variance (ANOVA) for continuous variables. Survival analyses were performed using the Kaplan-Meier method and compared using the log-rank test. Cox regression analyses were conducted to assess the relationship between the risk of recurrence and death with other prognostic factors. Statistical significance was defined as a p-value below 0.05.

## 3 Results

### 3.1 Patient population

**Figure 1:**
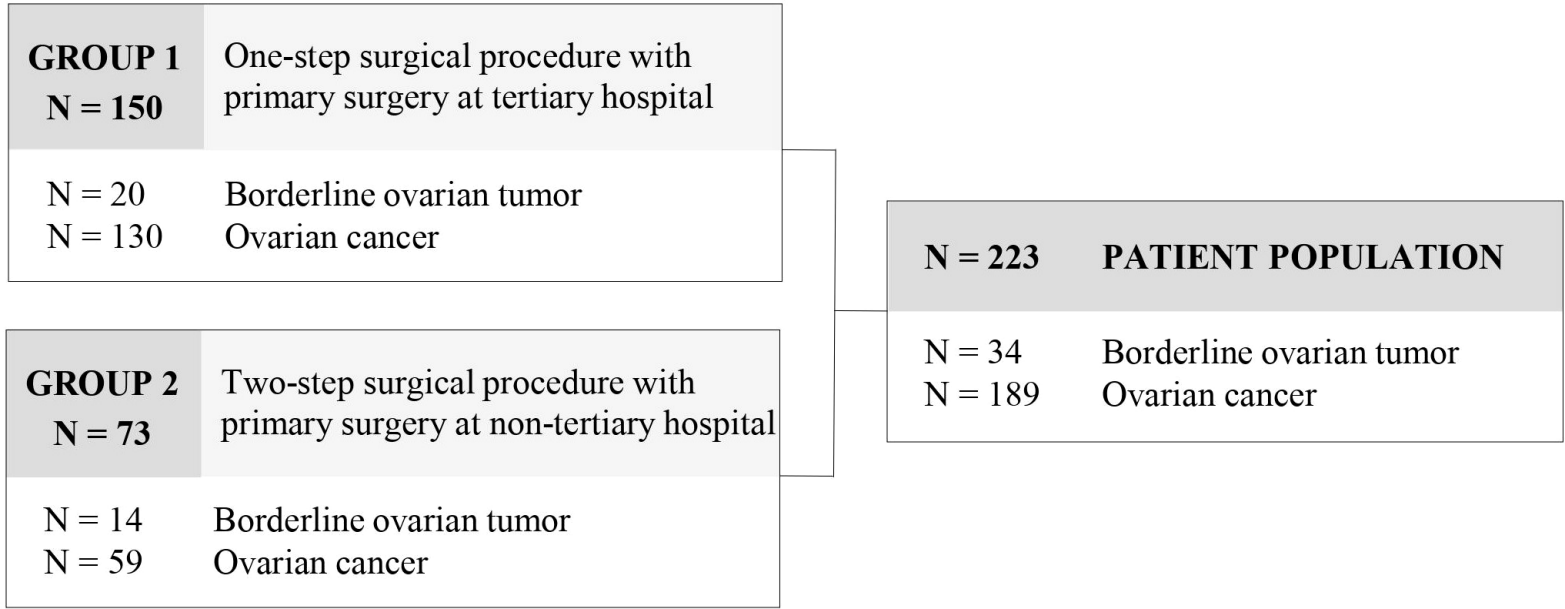
Subgroups of the patient population.

Out of 390 patients with adnexal masses, a total of 223 patients met the inclusion criteria and were enrolled in the study. 167 patients with benign histology or adnexal metastases of other origin were excluded from further analysis. 34 (15.2%) patients presented with a borderline ovarian tumor and 189 (84.8%) with ovarian cancer. Referral to our tertiary institution was preoperative in 150 patients due to suspected malignancy (group 1) and postoperative in 73 patients for completion of surgical staging and for adjuvant treatment after primary surgery at a non-tertiary center (group 2) (**Figure 1**). Mean age of the patient population was 59.1 (± 15.2) years and mean BMI was 25.0 (± 5.3) kg/m^2^. Clinicopathological data of the patient population are provided in **Table 1**. Patients in group 1 were significantly older at diagnosis (p=.031), exhibited more often ascites (p<.001), and presented with more advanced tumor stages (p=.038).

**Table 1:** Clinicopathological characteristics of the patient population.

	Patient population N = 223	Group 1: Primary surgery at tertiary gyn-onc center N = 150	Group 2: Primary surgery at non-tertiary center N = 73	P-value
Mean age at diagnosis, years ± SD	59.1 ± 15.2	60.6 ± 14.2	55.9 ± 16.9	**.031**
Mean BMI, kg/m^2^ ± SD	25.0 ± 5.3	25.0 ± 5.3	25.1 ± 5.3	.885
Menopausal status Premenopausal Perimenopausal Postmenopausal Missing	55 (24.7) 12 (5.4) 151 (67.7) 5 (2.2)	29 (19.3) 10 (6.7) 107 (71.3) 4 (2.7)	26 (35.6) 2 (2.7) 44 (60.3) 1 (1.4)	**.023**
Preoperative imaging, N (%) Vaginal ultrasound only Vaginal ultrasound + CT Vaginal ultrasound + MRI Vaginal ultrasound + CT + MRI	43 (19.3) 126 (56.5) 36 (16.1) 18 (8.1)	12 (8.0) 94 (62.7) 28 (18.7) 16 (10.7)	31 (42.5) 32 (43.8) 8 (11.0) 2 (2.7)	**<.001**
Imaging suspicious for malignancy, N (%)	164 (73.5)	134 (89.3)	30 (41.1)	**<.001**
Mean preoperative CA 125, kU/l ± SD	738 ± 1833	868 ± 2104	423 ± 810	.114
Ascites, N (%)	104 (46.6)	90 (60.0)	14 (19.2)	**<.001**
Localisation of adnexal mass, N (%) Unilateral Bilateral Unknown	144 (64.6) 68 (30.5) 11 (4.9)	90 (60.0) 54 (36.0) 6 (4.0)	54 (74.0) 14 (19.2) 5 (6.8)	**.029**
Dignity, N (%) Borderline ovarian tumor Ovarian cancer	34 (15.2) 189 (84.8)	20 (13.3) 130 (86.7)	14 (19.2) 59 (80.8)	.173
Histological subtype, N (%) Serous Mucinous Endometroid Clear cell Others	151 (67.7) 30 (13.5) 8 (3.6) 8 (3.6) 26 (11.7)	99 (66.0) 21 (14.0) 6 (4.0) 5 (3.3) 19 (12.7)	52 (71.2) 9 (12.3) 2 (2.7) 3 (4.1) 7 (9.6)	.634
Grading, N (%) Borderline Grade 1 Grade 2 Grade 3 Missing/not applicable	34 (15.2) 14 (6.3) 13 (5.8) 145 (65.0) 17 (7.6)	20 (13.3) 8 (5.3) 5 (3.3) 101 (67.3) 16 (10.7)	14 (19.2) 6 (8.2) 8 (11.0) 44 (60.3) 1 (1.4)	.056
FIGO stage, N (%) I II III IV Missing/not applicable	83 (37.2) 17 (7.6) 103 (46.2) 19 (8.5) 1 (0.4)	47 (31.3) 11 (7.3) 75 (50.0) 16 (10.7) 1 (0.7)	36 (49.3) 6 (8.2) 28 (38.4) 3 (4.1) 0 (0)	**.038**

A statistically significant p-value lower than 0.05 is marked bold in the table.

N, number; SD, standard deviation; BMI, body mass index; CT, computed tomography; MRI, magnetic resonance imaging; CA 125, Cancer-Antigen 125; FIGO, International Federation of Gynecology and Obstetrics.

### 3.2 Diagnostics

The diagnostic parameters compared between the two groups are detailed in **Table 1**. Patients with suspected malignancy and primary surgery at a tertiary center (group 1) underwent significantly more preoperative imaging than patients with incidental malignant findings and postoperative referral (group 2) (p<.001). In addition to vaginal ultrasound, 92.1% of patients in group 1 underwent additional CT or MRI, whereas 42.5% of patients in group 2 underwent only vaginal ultrasound as diagnostic imaging. Preoperative imaging was suspicious for malignancy in 89.3% of patients who underwent centralized primary surgery, significantly more than in 41.1% of patients who underwent primary surgery at a non-tertiary center (p<.001). Mean preoperative CA 125 levels tended to be higher in group 1 than in group 2 (868 ± 2104 kU/l vs. 423 ± 810 kU/l, p=.114).

### 3.3 Treatment

#### 3.3.1 Surgical cytoreduction

A detailed description of the surgical treatment characteristics among the different study groups is provided in **Table 2A**. Six patients were ineligible for primary surgical cytoreduction due to the extent of disease or the presence of a medical condition contraindicating general anesthesia or surgical intervention. Of the 217 patients who underwent surgical cytoreduction, complete cytoreduction was achieved in 203 (93.5%) patients, with equal distribution among the study subgroups (93.1% in group 1 vs. 94.4% in group 2, p=.913). Minimal residual disease (<1cm) was present in 11 (5.1%) patients and macroscopic residual disease (>1cm) in three (1.4%) patients, with no significant difference in residual disease between the two subgroups. Mean number of surgical interventions until completion of staging surgery was significantly higher in patients in group 2 compared to group 1 (2.1 vs. 1.3, p<.001). 99 (66%) patients in group 1 and 38 (52.1%) patients in group 2 underwent at least one laparotomy (p=.032). Mean time from diagnosis until completion of surgical cytoreduction was 31.9 days in group 1, and 73.7 days in group 2 (p<.001). Excluding patients treated with neoadjuvant chemotherapy, the mean time from diagnosis to completion of surgery was 1.9 days in group 1 and 34.7 days in group 2 (p<.001). There was neither a difference in intraoperative (p=.664) or postoperative (p=.884) complication rates, nor in cumulative duration of surgery (p=.609), cumulative blood loss (p=.159), or total hospitalization days (p=.369) between the study groups.

**Table 2A:** Characteristics of surgical treatment of the total patient population.

	Total N = 223	Group 1: Primary surgery at tertiary gyn-onc center N = 150	Group 2: Primary surgery at non-tertiary center N = 73	P-value
Mean number of surgical interventions, N ± SD	1.6 ± 0.6	1.3 ± 0.5	2.1 ± 0.3	**<.001**
Patients with at least one laparotomy, N (%)	137 (61.4)	99 (66)	38 (52.1)	**.032**
Mean number of hospitalization days, days ± SD	11.4 ± 6.3	11.1 ± 6.3	12.0 ± 6.2	.369
Surgical cytoreduction, N (%) Yes No	217 (97.3) 6 (2.7)	145 (96.7) 5 (3.3)	72 (98.6) 1 (1.4)	.666
Mean time from diagnosis until completion of surgical cytoreduction, days ± SD	45.9 ± 60.7	31.9 ± 56.7	73.7 ± 59.3	**<.001**
Cumulative duration of surgery, minutes ± SD	326.8 ± 137.1	329.4 ± 141.6	315.8 ± 116.9	.609
Cumulative blood loss, ml ± SD	430.7 ± 435.0	456.6 ± 412.3	350.2 ± 495.8	.159
Intraoperative complications, N (%)	27 (12.1)	17 (11.3)	10 (13.7)	.664
Postoperative complications, N (%)	85 (38.1)	58 (38.7)	27 (37.0)	.884
Residual disease, N (%) No cytoreduction performed Complete cytoreduction Minimal residual disease Macroscopic residual disease	6 (2.7) 203 (91.0) 11 (4.9) 3 (1.3)	5 (3.3) 135 (90.0) 8 (5.3) 2 (1.3)	1 (1.4) 68 (93.2) 3 (4.1) 1 (1.4)	.719

A statistically significant p-value lower than 0.05 is marked bold in the table. N, number; SD, standard deviation; ml, milliliter.

#### 3.3.2 Chemotherapy

A total of 171 patients with ovarian cancer were treated with chemotherapy, 79.3% patients in group 1 and 71.2% patients in group 2 (p=.182, **Table 2B**). Patients in group 1 received more adjuvant (54.7% vs. 30.1%, p<.001) and patients in group 2 more neoadjuvant (41.1% vs. 24.7%, p=.019) chemotherapy. Mean time from diagnosis of ovarian cancer until start of chemotherapy was longer in patients in group 2 than in group 1 (45.1 vs. 33.3 days, p=.005). This difference was most evident in patients treated with adjuvant chemotherapy (39.2 days in group 1 compared to 67.8 days in group 2, p<.001), but still present in patients undergoing neoadjuvant systemic treatment (21.1 days in group 1 compared to 29.9 days in group 2, p=.027). Mean time from completion of surgical cytoreduction until start of adjuvant chemotherapy showed no difference between the study subgroups (p=.455).

**Table 2B:** Characteristics of adjuvant treatment for patients diagnosed with ovarian cancer.

	Total N = 189	Group 1: Primary surgery at tertiary gyn-onc center N = 130	Group 2: Primary surgery at non-tertiary center N = 59	P-value
Adjuvant treatment, N (%) No chemotherapy Adjuvant chemotherapy Neoadjuvant chemotherapy	18 (9.5) 104 (55.0) 67 (35.4)	11 (8.5) 82 (63.1) 37 (28.5)	7 (11.9) 22 (37.3) 30 (50.8)	**.004**
Mean time from diagnosis until start of chemotherapy, days ± SD	37.0 ± 24.6	33.3 ± 17.7	45.1 ± 34.3	**.005**
Mean time from completion of surgical cytoreduction until start of adjuvant chemotherapy, days ± SD	37.2 ± 16.0	37.9 ± 16.6	34.9 ± 13.6	.455

A statistically significant p-value lower than 0.05 is marked bold in the table.

N, number; SD, standard deviation; ml, milliliter.

### 3.4 Clinical outcome

After a mean follow-up of 62.2 (95% CI 58.2-66.3) months, 92 (41.3%) patients experienced at least one recurrence, and 81 (36.3%) patients died. Recurrence rates were 8.8% (3/34) in patients diagnosed with borderline ovarian tumor, 25.4% (17/67) in patients with early-stage ovarian cancer, and 59.5% (72/121) in patients with advanced-stage ovarian cancer. Mean recurrence-free survival was 75.2 (95% CI 66.6-83.9) months and mean overall survival was 85.4 (95% CI 76.7-94.1) months for the entire patient population. Among patients with borderline ovarian tumor, mean recurrence-free survival was 108.4 (95% CI 91.2-125.6) months, with no significant difference between group 1 (107.6 months, 95% CI 84.1-131.1) and group 2 (106.6 months, 95% CI 80.4-132.9) (log-rank, p=.472). In patients with early-stage ovarian cancer, mean recurrence-free survival was 85.0 (95% CI 74.1-95.8) months, with 72.1 (95% CI 59.5-84.7) months in group 1 and 94.1 (95% CI 79.2-108.9) months in group 2 (log-rank, p=.216). Patients with advanced stage ovarian cancer showed a mean recurrence-free survival of 50.4 (95% CI 39.9-60.9) months, with 35.4 (95% CI 30.0-40.9) months in group 1 and 56.3 (95% CI 35.7-76.9) months in group 2 (log-rank, p=.833). Incidental diagnosis of borderline ovarian tumor or ovarian cancer with postoperative referral to a tertiary center for completion of surgical treatment was not associated with increased risk of recurrence (HR 0.8, 95% CI 0.6-1.8, p=.839) or death (HR 0.7, 95% CI 0.4-1.1, p=.113) in univariable Cox regression analysis. Also, in multivariable Cox regression analysis including dignity, stage and age at diagnosis, there was no increased risk of recurrence (HR 1.02, 95% CI 0.64-1.61, p=0.948) or death (HR 0.81, 95%-CI 0.49-1.34, p=0.41) for patients in group 2.

## 4 Discussion

### 4.1 Summary of main results

This retrospective cohort study evaluated the surgical and oncologic outcomes of patients who were referred to our tertiary hospital preoperatively with suspected malignancy and patients who underwent surgery at a non-tertiary hospital and were referred to our tertiary hospital postoperatively for complete surgical staging and adjuvant treatment after incidental findings of malignancy. There were no differences between the study subgroups in the number of complete cytoreductions, intraoperative and postoperative complications, cumulative duration of surgery, cumulative blood loss, or total number of hospital days. However, patients with incidental malignancy findings and postoperative referral underwent more surgical procedures and experienced a delay in therapy with a longer time interval from diagnosis to completion of surgical cytoreduction and to initiation of chemotherapy. Nevertheless, incidental diagnosis of borderline ovarian tumor or ovarian cancer with a two-stage surgical procedure and postoperative referral was not associated with an increased risk of recurrence or death.

### 4.2 Results in the context of published literature

In ovarian cancer, there is strong evidence that centralization is associated with better quality of care and prolonged survival (3–8). Several studies have shown that complete cytoreduction is best performed by gynecologic oncologists (4–6, 8, 15) and that maximal cytoreduction is one of the strongest determinants of survival in patients with ovarian cancer (16–18). In our study, a two-step procedure with diagnostic surgery in a non-tertiary hospital followed by surgical cytoreduction in a tertiary hospital resulted in the same rates of complete cytoreduction as a single-stage centralized procedure with intraoperative diagnosis of malignancy.

A population-based cohort study has further shown that centralization can significantly shorten the time interval between primary surgical cytoreduction and chemotherapy (8), but it is controversial whether this time interval affects prognosis in ovarian cancer. Some studies have shown that delayed initiation of chemotherapy after surgery is associated with poorer prognosis in patients with advanced ovarian cancer (19, 20), whereas other studies have found no such association (21, 22). In our study, the two subgroups showed no difference in the time interval between surgical cytoreduction and initiation of chemotherapy, indicating that this duration is not affected by incidental malignancy findings. However, excluding patients treated with neoadjuvant chemotherapy, group 2 had a significantly longer time interval between diagnosis and completion of surgical cytoreduction. According to current literature, a delay of staging surgery is inevitable in case of totally unexpected malignancy findings in final pathology (23). As a delay between laparoscopy and laparotomy of more than 17 days could adversely affect the distribution of disease stage it should however be kept to a minimum (24). Therefore, timely centralization with re-evaluation and definitive surgery should be sought in these patients.

According to our study results, patients who underwent centralization postoperatively experienced a significantly higher number of surgical procedures, yet this increase did not lead to higher rates of intraoperative or postoperative complications or longer hospital stays. The question of whether a two-stage surgical approach negatively affects patient outcomes is controversial, and conclusive data are lacking. Demir et al. (11) suggested that a two-stage procedure for women with ovarian cancer does not have an adverse effect on patients, provided appropriate steps are followed during the initial surgery, which is consistent with our findings.

According to the current literature, centralization is conclusively associated with a lower risk of recurrence and better survival for patients with ovarian cancer (4–6, 8). However, our study subgroups did not differ in the risk of recurrence or death and showed comparable mean recurrence-free survival. Therefore, incidental postoperative diagnosis of a borderline ovarian tumor or ovarian cancer with subsequent centralized second surgery does not appear to have a negative impact on patient survival.

### 4.3 Strengths and weaknesses

To our knowledge, this is the first study to examine the surgical and oncological outcomes of patients with malignant adnexal masses depending on whether they were referred to a tertiary hospital for suspected malignancy before histological diagnosis or after incidental findings of malignancy. A major strength of our study is the long follow-up period and centralized pathological review. The main methodological limitation of our study is the risk of bias due to its retrospective nature. As the study subgroups differed in terms of age, dignity and stage, other unrecorded patient and tumor characteristics may have influenced treatment and outcome, despite statistical adjustments to account for these systematic differences. In addition, the histological heterogeneity of borderline and highly malignant ovarian tumors in this cohort limits the interpretation of chemotherapy and combined treatment approaches with interval debulking surgery. Furthermore, the results of our study are of limited statistical power due to the relatively small sample size, especially in patients with borderline ovarian tumors.

### 4.4 Implications for practice and future research

To ensure appropriate initial disease management for a disease as insidious and deadly as ovarian cancer, it is recommended that all patients with adnexal masses suspected of malignancy be referred directly to a gynecologic oncologist (12, 25, 26). However, centralization may not always occur in a timely manner, as the insufficient sensitivity of preoperative diagnostics compromises the ability to adequately triage patients with adnexal masses (10, 11). Since ovarian cysts are very common, surgery of all patients with adnexal masses by oncologic gynecologists would not be reasonable. Consequently, not every woman with an adnexal mass can be referred to a specialized hospital prior to the diagnosis of ovarian cancer, and general gynecologists will continue to operate on women in whom malignancy is not detected until postoperative final pathology. In this regard, our study showed that patients with an incidental postoperative diagnosis of borderline ovarian tumor or ovarian cancer in a non-tertiary hospital do not have impaired oncological outcomes if the diagnosis of malignancy is followed by direct referral to and multidisciplinary review by a tertiary hospital. Therefore, until highly sensitive preoperative diagnostics with correct triaging of all patients with adnexal masses is achieved, a two-stage surgical procedure with centralization after diagnostic surgery could be considered a reasonable alternative for patients with adnexal masses of uncertain malignancy. In addition, such a two-stage surgical approach could reduce the risk of overtreatment and anxiety in patients with adnexal masses, allow discussion of pathology reports prior to surgical cytoreduction, and address concerns about future fertility. Careful intraoperative and postoperative management of all patients with adnexal masses by general gynecologists would become more important, and optimization strategies for guideline-based primary care should be sought as part of future research (27).

## 5 Conclusion

Patients with adnexal masses and unexpected findings of borderline ovarian tumor or ovarian cancer at a non-tertiary hospital with postoperative referral to a tertiary hospital for completion of surgical staging and adjuvant treatment showed no increased risk of recurrence or death compared with patients with suspected malignancy and centralized single-stage surgery. There were no differences in the rates of complete cytoreduction and complications, but incidental findings of malignancy followed by a second surgery resulted in a higher number of surgical procedures and a delay in initiation of therapy after diagnosis.

## Data Availability

The raw data supporting the conclusions of this article will be made available by the authors, without undue reservation.

## References

[1] Sung H Ferlay J Siegel RL Laversanne M Soerjomataram I Jemal A. Global cancer statistics 2020: GLOBOCAN estimates of incidence and mortality worldwide for 36 cancers in 185 countries. CA Cancer J Clin. (2021) 71:209–49. doi: 10.3322/caac.21660 33538338

[2] Momenimovahed Z Tiznobaik A Taheri S Salehiniya H. Ovarian cancer in the world: Epidemiology and risk factors. Int J Womens Health. (2019) 11:287–99. doi: 10.2147/IJWH.S197604 PMC6500433 31118829

[3] Querleu D Planchamp F Chiva L Fotopoulou C Barton D Cibula D. European society of gynaecologic oncology quality indicators for advanced ovarian cancer surgery. Int J Gynecol Cancer. (2016) 26:1354–63. doi: 10.1097/IGC.0000000000000767 27648648

[4] Woo YL Kyrgiou M Bryant A Everett T Dickinson HO. Centralization of services for gynaecological cancers - A Cochrane systematic review. Gynecol Oncol. (2012) 126:286–90. doi: 10.1016/j.ygyno.2012.04.012 22507534

[5] Palmqvist C Staf C Mateoiu C Johansson M Albertsson P Dahm-Kähler P. Increased disease-free and relative survival in advanced ovarian cancer after centralized primary treatment. Gynecol Oncol. (2020) 159:409–17. doi: 10.1016/j.ygyno.2020.09.004 32943206

[6] Vernooij F Heintz P Witteveen E van der Graaf Y. The outcomes of ovarian cancer treatment are better when provided by gynecologic oncologists and in specialized hospitals: A systematic review. Gynecol Oncol. (2007) 105:801–12. doi: 10.1016/j.ygyno.2007.02.030 17433422

[7] Fung-Kee-Fung M Kennedy EB Biagi J Colgan T D’Souza D Elit LM. The optimal organization of gynecologic oncology services: A systematic review. Curr Oncol. (2015) 22:e282–93. doi: 10.3747/co.22.2482 PMC4530826 26300679

[8] Dahm-Kähler P Palmqvist C Staf C Holmberg E Johannesson L. Centralized primary care of advanced ovarian cancer improves complete cytoreduction and survival - A population-based cohort study. Gynecol Oncol. (2016) 142:211–6. doi: 10.1016/j.ygyno.2016.05.025 27238084

[9] Olaitan A McCormack M. Centralization of services for the management of ovarian cancer: Arguments for. BJOG. (2007) 114:1188–90. doi: 10.1111/j.1471-0528.2007.01460.x 17877670

[10] Matsushita H Watanabe K Yokoi T Wakatsuki A. Unexpected ovarian Malignancy following laparoscopic excision of adnexal masses. Hum Reproduction. (2014) 29:1912–7. doi: 10.1093/humrep/deu162 24964925

[11] Demir RH Marchand GJ. Adnexal masses suspected to be benign treated with laparoscopy. J Soc Laparoendoscopic Surgeons. (2012) 16:71–84. doi: 10.4293/108680812X13291597716069 PMC3407461 22906334

[12] Carvalho JP Moretti-Marques R Filho ALDS. Adnexal mass: Diagnosis and management. Rev Bras Ginecol e Obstetricia. (2020) 42:438–44. doi: 10.1055/s-0040-1715547 PMC10316833 32736396

[13] Colombo N Sessa C Du Bois A Ledermann J McCluggage WG McNeish I. ESMO-ESGO consensus conference recommendations on ovarian cancer: Pathology and molecular biology, early and advanced stages, borderline tumours and recurrent disease. Ann Oncol. (2019) 30:672–705. doi: 10.1093/annonc/mdz062 31046081

[14] Deutsche Krebsgesellschaft Deutsche Krebshilfe, AWMF. Leitlinienprogramm Onkologie: S3-Leitlinie Diagnostik, Therapie und Nachsorge Maligner Ovarialtumoren (2022). Available online at: https://www.leitlinienprogramm-onkologie.de/leitlinien/ovarialkarzinom/ (Accessed April 20, 2024).

[15] Giede KC Kieser K Dodge J Rosen B. Who should operate on patients with ovarian cancer? An evidence-based review. Gynecol Oncol. (2005) 99:447–61. doi: 10.1016/j.ygyno.2005.07.008 16126262

[16] Chang SJ Hodeib M Chang J Bristow RE. Survival impact of complete cytoreduction to no gross residual disease for advanced-stage ovarian cancer: A meta-analysis. Gynecol Oncol. (2013) 130:493–8. doi: 10.1016/j.ygyno.2013.05.040 23747291

[17] Bois AD Reuss A Pujade-Lauraine E Harter P Ray-Coquard I Pfisterer J. Role of surgical outcome as prognostic factor in advanced epithelial ovarian cancer: A combined exploratory analysis of 3 prospectively randomized phase 3 multicenter trials: by the arbeitsgemeinschaft gynaekologische onkologie studiengruppe ovarialkarzinom (AGO-OVAR) and the groupe d’Investigateurs nationaux pour les etudes des cancers de l’Ovaire (GINECO). Cancer. (2009) 115:1234–44. doi: 10.1002/cncr.v115:6 19189349

[18] Bristow RE Tomacruz RS Armstrong DK Trimble EL Montz FJ. Survival effect of maximal cytoreductive surgery for advanced ovarian carcinoma during the platinum era: a meta-analysis. J Clin Oncol. (2002) 20:1248–59. doi: 10.1200/JCO.2002.20.5.1248 11870167

[19] Hofstetter G Concin N Braicu I Chekerov R Sehouli J Cadron I. The time interval from surgery to start of chemotherapy significantly impacts prognosis in patients with advanced serous ovarian carcinoma - Analysis of patient data in the prospective OVCAD study. Gynecol Oncol. (2013) 131:15–20. doi: 10.1016/j.ygyno.2013.07.086 23877013

[20] Tewari KS Java JJ Eskander RN Monk BJ Burger RA. Early initiation of chemotherapy following complete resection of advanced ovarian cancer associated with improved survival: NRG oncology/gynecologic oncology group study. Ann Oncol. (2016) 27:114–21. doi: 10.1093/annonc/mdv500 PMC4684156 26487588

[21] Gadducci A Sartori E Landoni F Zola P Maggino T Maggioni A. Relationship between time interval from primary surgery to the start of taxane- plus platinum-based chemotherapy and clinical outcome of patients with advanced epithelial ovarian cancer: Results of a multicenter retrospective Italian study. J Clin Oncol. (2005) 23:751–8. doi: 10.1200/JCO.2005.03.065 15613698

[22] Paulsen T Kærn J Kjærheim K Haldorsen T Tropé C. Influence of interval between primary surgery and chemotherapy on short-term survival of patients with advanced ovarian, tubal or peritoneal cancer. Gynecol Oncol. (2006) 102:447–52. doi: 10.1016/j.ygyno.2006.01.035 16516277

[23] Muzii L Angioli R Zullo M Panici PB. The unexpected ovarian Malignancy found during operative laparoscopy: Incidence, management, and implications for prognosis. J Minim Invasive Gynecol. (2005) 12:81–9. doi: 10.1016/j.jmig.2004.12.019 15904606

[24] Lehner R Wenzl R Sevelda P. Influence of delayed staging laparotomy after laparoscopic removal of ovarian masses later found Malignant. Obstetrics Gynecol. (1998) 92:967–71. doi: 10.1016/s0029-7844(98)00323-8 9840559

[25] Glanc P Benacerraf B Bourne T Brown D Coleman BG Crum C. First international consensus report on adnexal masses: management recommendations. J Ultrasound Med. (2017) 36:849–63. doi: 10.1002/jum.14197 28266033

[26] Salvador S Scott S Glanc P Eiriksson L Jang JH Sebastianelli A. Guideline no. 403: initial investigation and management of adnexal masses. J Obstetrics Gynaecol Canada. (2020) 42:1021–9. doi: 10.1016/j.jogc.2019.08.044 32736853

[27] Marano L Verre L Carbone L Poto GE Fusario D Venezia DF. Current trends in volume and surgical outcomes in gastric cancer. Vol. 12. J Clin Med Multidiscip Digital Publishing Institute (MDPI). (2023) 12:2708. doi: 10.3390/jcm12072708 PMC10094776 37048791

